# Characteristics of alpha-1 antitrypsin deficiency related lung disease exacerbations using a daily symptom diary and urinary biomarkers

**DOI:** 10.1371/journal.pone.0297125

**Published:** 2024-02-02

**Authors:** Paul Ellis, Gita Parekh, Annelyse Duvoix, Lynne Watson, Alex Sharp, Farah Mobeen, Anita Pye, Robert Stockley, Alice Turner

**Affiliations:** 1 Institute of Applied Health Research, University of Birmingham, Birmingham, United Kingdom; 2 University Hospitals Birmingham NHS Foundation Trust, Birmingham, United Kingdom; 3 Mologic Ltd, Thurleigh, Bedford, United Kingdom; 4 Institute of Inflammation and Aging, University of Birmingham, Birmingham, United Kingdom; Medizinische Fakultat der RWTH Aachen, GERMANY

## Abstract

**Background:**

Pulmonary exacerbations in alpha-1 antitrypsin deficiency (AATD) related lung disease are a significant contributor to disease burden, as with usual COPD. Separating the early stages of an exacerbation from the day-to-day variation in stable COPD is central to the concerns of both clinicians and patients and has been identified as a research priority by NIHR. Clinical tools that distinguish baseline symptoms from those of an exacerbation could allow early and appropriate treatment of AECOPD to reduce the impact and potentially may slow disease progression thereby improving survival and quality of life. Candidate tools include symptom diaries and biomarkers of infection and acute inflammation. Urinary biomarkers of AECOPD have yet to be explored in AATD related COPD.

**Methods:**

55 patients with AATD related lung disease with a history of 2 or more AECOPD in the preceding year were prospectively followed for 18 months. Each patient recorded symptom scores daily via an electronic symptom diary (eDiary) based on Bronkotest. Urinary biomarkers for AAT, NE, CRP, TIMP1 and desmosine were measured weekly using a home urinary lateral flow device. During self-reported AECOPD patients were asked to perform urine analysis on the first 7 consecutive days.

**Results:**

Type I Anthonisen exacerbations and episodes occurring in autumn/winter lasted longer than Type II/III exacerbations and spring/summer episodes respectively. Median urinary CRP concentration across all study participants increased during Type I AECOPD. eDiary adherence was 68% over a median of 17.8 months (IQR 15.7 to 18.5).

**Conclusions:**

Use of an eDiary and urinary biomarkers to detect and characterise AECOPD remotely in AATD related lung disease is feasible over a prolonged period and paves the way for precision detection of exacerbations.

## Introduction

Alpha-1 antitrypsin deficiency (AATD) is an autosomal-codominant condition that predisposes to development of pulmonary emphysema, typically in a panlobular bibasal distribution. AATD related lung disease often presents earlier and with more modest smoking histories, than usual COPD.

Pulmonary exacerbations of COPD in AATD contribute significantly to symptoms and quality of life (QOL) [1]. As with usual COPD, the heterogenous nature of the presentation and aetiology of such episodes makes developing targeted therapies challenging. Indeed, there have been no new therapies for treatment of acute exacerbations of COPD (AECOPD) or AATD related lung disease for more than thirty years.

A recent research prioritisation exercise established ten research priorities in the field of COPD exacerbations [2]. Two of the highest ranked priorities were ‘What is the best way to tell the start of an exacerbation from day-to-day variation in symptoms?’ and ‘What is the best way to tell the difference between an exacerbation and a different cause of changing symptoms in a person with COPD?’. Cohort studies focusing on biomarkers fit this brief, since specific biomarker(s), in combination with a validated symptom score, could enable more precise identification and treatment of exacerbations [3]. For patients with AATD, where emphysema and physiological decline is more rapid, this may slow the rate of emphysema progression by reducing the associated inflammation [4].

Until recently, AECOPD were defined by symptom-based definitions or healthcare use, especially in clinical trials. Event-based definitions are indirect measures of exacerbations and do not include similar episodes that remain untreated (“unreported”) which may be twice as common [4, 5]. More recently, patient reported outcome (PRO) tools have been developed to help characterise and quantify symptoms during exacerbations [6], largely in the context of randomised controlled trials where follow up is intensive and adherence constantly monitored. These trials have also focused on usual COPD and not AATD related lung disease where inflammation [7] and duration [8] of the episodes are greater. Few studies have validated PRO tools in this population [9]. One exception is Bronkotest [9, 10] which, like other PRO scores for exacerbations [6], incorporates domains of the usual exacerbation symptoms in addition to “well-being” into a quantifiable symptom score [10].

Urinary biomarkers have yet to be explored in depth for AECOPD. Urine analysis is a simple and non-invasive way of measuring excretion of systemic biomarkers and is not dependent on sputum production and assessment. Elastin degradation pathways have showed promise as candidate biomarkers of disease activity and exacerbations [11–13], in addition to more conventional measures of inflammation [14].

The current study aimed to characterise AECOPD in frequent exacerbators with AATD related lung disease using an eDiary and a point of care (POC) urinary biomarker measurement device. We set out to explore the relationship of Anthonisen defined exacerbations with changes in urinary biomarkers of elastin degradation and inflammation and hypothesised the concentration of such biomarkers would increase during an AECOPD and return toward baseline value upon symptom recovery. We also aimed to determine if such enhanced monitoring of patients could accurately predict Anthonisen defined AECOPD events compared with physician assessment of symptoms alone. Feasibility of eDiary and POC urine monitors was determined by their implementation into real-world patients without the intensive follow up typical of randomised controlled trials. This should be informative for development and roll out of medical devices or patient facing tools which aid AECOPD recognition and management.

## Methods

This analysis reviewed daily symptom diary data over an 18-month period with regular monitoring of urinary biomarkers from a cohort of 55 severe AATD patients recruited to the Elastin degradation in exacerbations of AATD related lung disease study (REC 16/WA/0352, approved by the Health and Care Research Wales). Written consent was gained for all participants. Each patient had a primary diagnosis of COPD confirmed with spirometry (FEV_1_/FVC ratio <0.7/LLN) and received standard COPD care in line with the GOLD strategy [1]. Patients with a history and diagnosis of asthma were excluded.

Participants were recruited between January 2017 and March 2018 from AATD clinics and registry in Birmingham, UK. The eDiary device (Kindle Fire HD 8", sixth generation) and a POC urine lateral flow assay device (Headstart®, Mologic, Bedfordshire, UK) were issued to patients during the baseline visit with appropriate training. Each eDiary device was preloaded with the Take Part application, an electronic form of the validated paper Bronkotest diary [9]. S1 Table in S1 File provides a breakdown of the symptom score domains and weighting. Patients were asked to complete the eDiary daily.

Participants were instructed to use the POC urine lateral flow assay device (known as a ‘urine cube’) on a weekly basis during periods of stable symptoms and for 7 consecutive days from the first day of an exacerbation. The urine cube collected and stored optical density (OD) data from a lateral flow assay measuring alpha-1 antitrypsin (AAT), neutrophil elastase (NE), tissue inhibitor of metalloproteinases 1 (TIMP1), C-reactive Protein (CRP) and desmosine. DataReader Software was used to extract measurements from the urine cube at study visits and on completion. Upon study completion, participants completed a short questionnaire assessing the usability of the urine cube.

### Data analysis

To maximise the value of the symptom data collected, imputation was used, as in previous studies analysing symptom diary data [15]; see also S1 Fig in S1 File. The imputation algorithm adhered to the following rules (i) if ≤2 days of data were missing the most recently available data was used for imputation; (ii) any data not recorded as part of 3 or more consecutive days was not imputed and was recorded as missing. This was reset once there were 5 consecutive days of stable symptoms to ensure an exacerbation was not missed or included in stable data fields. Symptom and treatment data were analysed for each patient separately. Exacerbations were defined by Anthonisen criteria [16] with two days of worsening symptoms including one or more of increased breathlessness, new or increased sputum production and new or increased sputum purulence. Treated episodes outside this definition were recorded as “unclassified”. Use of antibiotics or steroids for indications other than pulmonary exacerbations (e.g., urinary tract infection) were also excluded. Resolution of an exacerbation was achieved if symptoms returned to baseline for ≥5 days with the resolution day defined as the first of 5 consecutive stable days. Any exacerbations that had incomplete data were censored at the point where data was missing until symptoms had returned to baseline.

OD data collected from the urine cube device at the end of the study was transformed to analyte concentrations calculated from concentration curves by Mologic.

### Statistical approach

Baseline characteristics are presented as means and standard deviation for normally distributed continuous variables. Medians and interquartile ranges are presented for non-normally distributed variables.

Exacerbation characteristics were compared using the ‘tableone’ package in R Studio [17]. For continuous variables, t-tests were used for normally distributed data and the Kruskal-Wallis test for non-normally distributed data. Chi-squared tests were used to compare categorical variables. Normality of continuous variables were determined by comparison of mean, median and their respective variances, histograms, Q-Q plots and Kolmogorov Smirnov tests of normality.

Average symptom score data by day were compared visually using mean symptom score for Anthonisen type, treated vs untreated exacerbations and seasons defined as: autumn/winter (October to March), spring/summer (April to September).

Receiver operator characteristic (ROC) curves were constructed to explore the diagnostic ability of the symptom score for Type I exacerbations across a range of definitions for change in symptom score (daily change, 3-day rolling mean and 7-day rolling mean, S4 Fig in S1 File). Sensitivity and specificity and impact of missing values was explored; the optimum was 7-day rolling mean.

Percentage completion of the eDiary was used as a marker of adherence and feasibility. Data on eDiary usability was collected.

To track urinary biomarkers throughout the course of an exacerbation including the pre-exacerbation phase, concentrations of AAT, NE, TIMP1, CRP and desmosine were compared across 5 distinct periods; 14 days to -7 days, -7 days to 0 days, 0 days to 3 days, 3 days to 4 days and >7 days. A sub analysis of Type I exacerbations vs Type II/III exacerbations was also performed.

Thematic analysis of the end of study questionnaire free text responses was used to qualitatively assess the usability of the urine cube device.

## Results

Baseline characteristics of all patients are in Table 1.

**Table 1:** Patient characteristics.

n	55
**Female (%)**	21 (38.9)
**Age**	55.14 ±9.13
**BMI**	24.64 [22.48, 30.42]
Smoking status (%)	
Never smoker	6 (11.1)
Ex-smoker	46 (85.2)
Current smoker (%)	2 (3.7)
**Pack year history**	19.00 [8.00, 25.00]
**FEV**_**1**_ **/L**	1.24 [1.00, 1.68]
**FEV**_**1**_ **% predicated**	41.40 ±16.01
**FVC/L**	3.73 ±1.13
**FVC % predicted**	89.74 ±22.90
**FEV**_**1**_ **/FVC ratio**	0.37 ±0.11
**KCO % predicted**	57.02 ±16.62
**MRC score (%)**	
0	1 (2.0)
1	6 (12.0)
2	13 (26.0)
3	25 (50.0)
4	5 (10.0)
**CAT score**	27.00 [20.00, 30.00]
**SGRQ total**	65.39 [53.06, 75.03]
**Annual exacerbation rate**	3.00 [2.00, 3.25]
**Oxygen therapy (%)**	5 (9.3)

Data are expressed as frequency (%), mean +/-SD or median [1^st^ quartile, 3^rd^ quartile]. %; percentage, BMI; body mass index, FEV_1_; forced expiratory volume in 1 second, FVC; forced vital capacity, KCO; diffusion capacity for carbon monoxide MRC; Medical Research Council, CAT; COPD assessment test, SGRQ; St Georges Respiratory Questionnaire at baseline.

Overall, adherence was 68% for daily symptom diary testing over a median follow up of 17.8 months (IQR 2.8). For those with >50% adherence (n = 38) this increased to 76.1% with a median follow up period of 13.6 months (IQR 7.1). A sensitivity analysis was performed that excluded patients with less than 90 entries (representing ≥3 months of data) to remove noncompliers or those using the eDiary for a short period. In this group, 49% of data was missing suggesting compliance decreased the longer the patient used the eDiary. The majority of patients scored the device as “very easy” or “easy”.

### AECOPD characteristics

**Fig 1:**
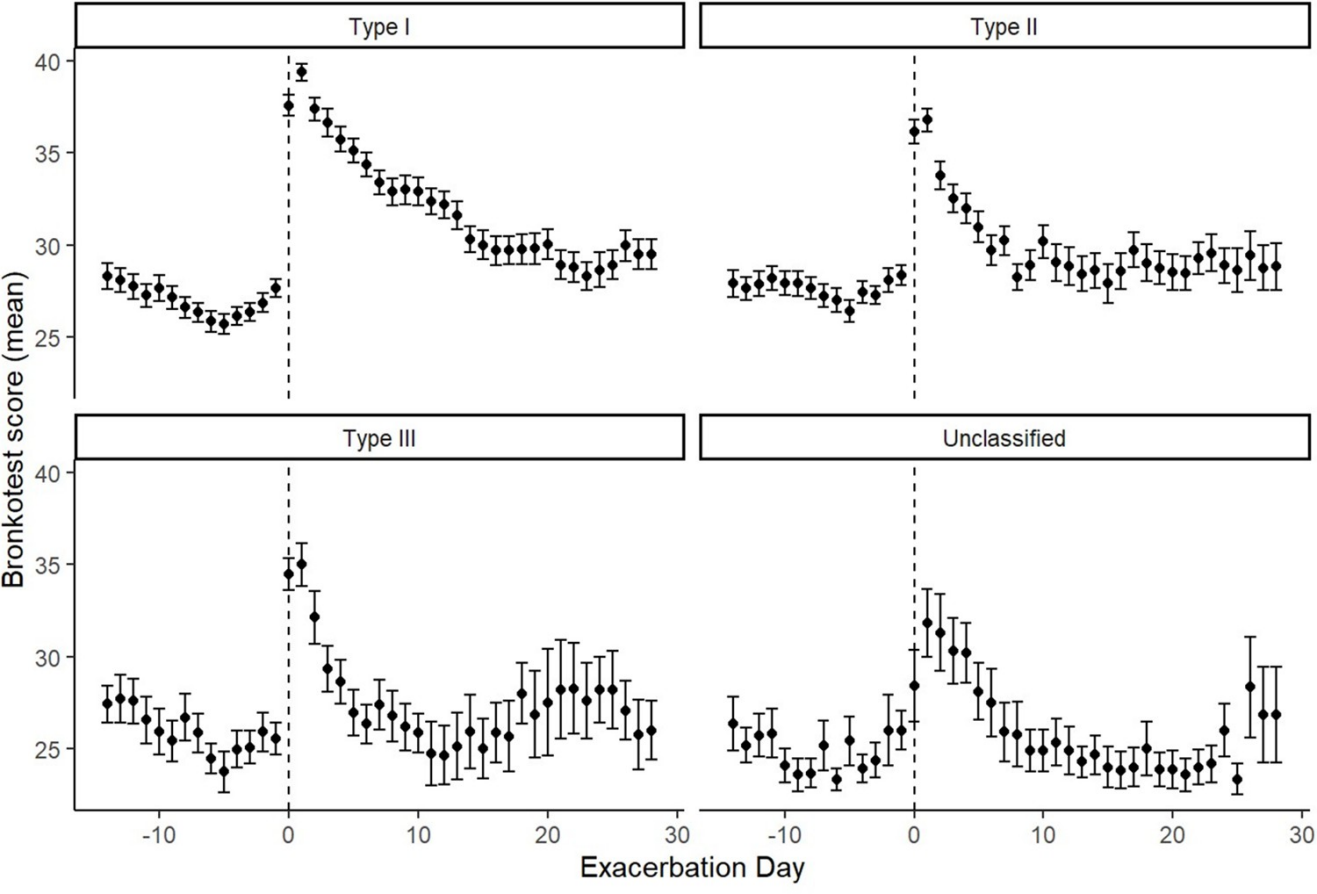
Bronkotest score during exacerbations by Anthonisen type. Unclassified exacerbations were treated episodes that did not meet Anthonisen criteria.

**Fig 2:**
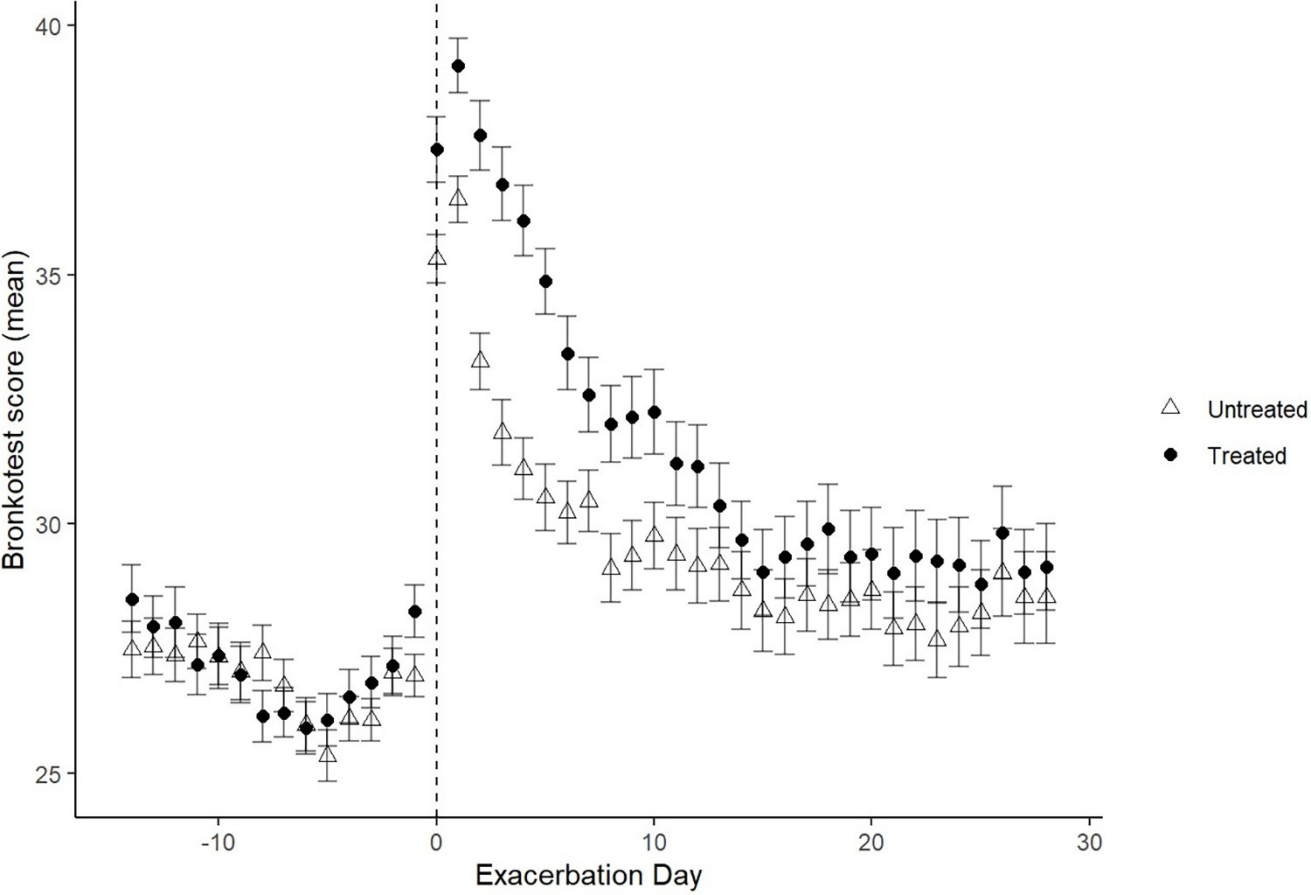
Bronkotest score during treated and untreated exacerbations.

There were 271 Anthonisen defined and 13 unclassified exacerbations (Table 2). The majority (51.7%) were Type I which were longer in duration than Type II and Type III (median difference 6 days, p < 0.001). There was no difference in treatment delay between episode types. The peak Bronkotest score during a Type I exacerbation was higher than Type II and Type III exacerbations (Fig 1). Fig 2 shows the average Bronkotest score at different time points before and after a treated or untreated exacerbation. There was no difference in symptom score in the 14 days before an exacerbation, followed by a significantly higher symptom score in treated episodes on the day prior to a symptom defined exacerbation. For both treated and untreated episodes the peak tended to be on the second day of an exacerbation and was significantly higher (mean difference 4.0 points, p < 0.001) in the treated episodes. Symptoms recovered back to baseline quicker (p <0.001) in untreated episodes (median duration 5.00 days IQR 9.0) compared to treated episodes (median 8.00 days IQR 13.0).

**Table 2:** Comparison of Type I vs Type II/III Anthonisen defined exacerbations in patients with AATD related lung disease.

	Type I	Type II/III	*P*
**n**	140	131	
**Season (%)**			0.217
Autumn	41 (29.5)	38 (29.0)	
Spring	31 (22.3)	32 (24.4)	
Summer	23 (16.5)	32 (24.4)	
Winter	44 (31.7)	29 (22.1)	
**Duration (days)**	10.00 [5.00, 22.50]	4.00 [2.00, 9.00]	<0.001
**Severity (%)**			<0.001
Mild	62 (44.6)	93 (71.0)	
Moderate	72 (51.8)	34 (26.0)	
Severe	5 (3.6)	4 (3.1)	
**Treatment delay (days)**	1.00 [0.00, 4.00]	0.00 [0.00, 2.00]	0.074
**Peak symptom score**	44.65 (5.43)	39.52 (5.76)	<0.001

Data are expressed as frequency (%), mean +/-SD or median [1^st^ quartile, 3^rd^ quartile]. Seasons are defined as: autumn/winter (October to March), spring/summer (April to September). Exacerbation severity: mild; bronchodilators only, moderate; oral steroids, antibiotics or both, severe; hospital admission. %; percentage, BMI; body mass index, FEV_1_; forced expiratory volume in 1 second, FVC; forced vital capacity, KCO; diffusion capacity for carbon monoxide MRC; Medical Research Council, CAT; COPD assessment test, SGRQ; St Georges Respiratory Questionnaire at baseline

**Fig 3:**
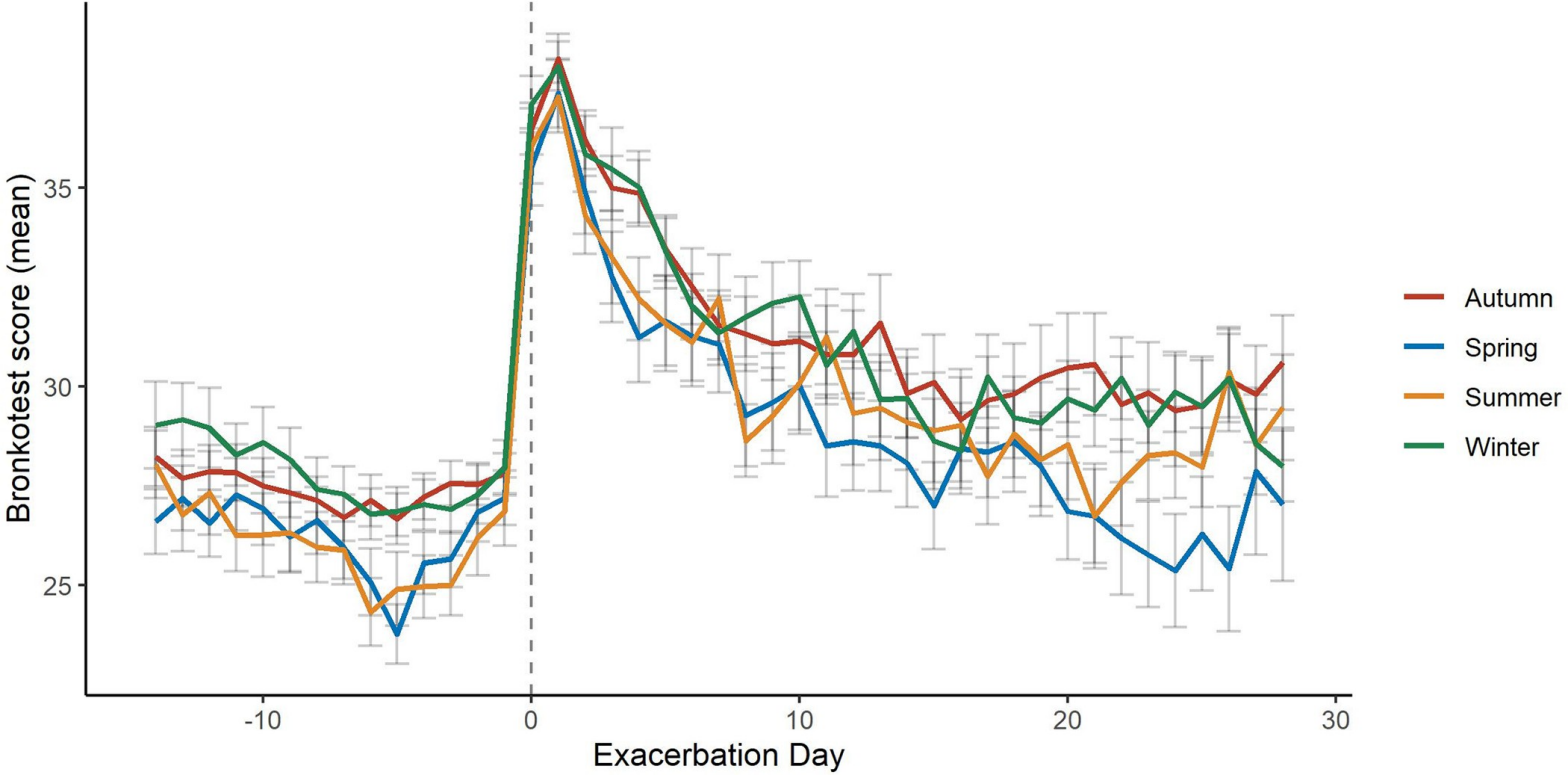
Bronkotest score during exacerbations in different seasons of the year.

Of 125 treated exacerbations, 106 (84.8%) were associated with both feeling generally worse than usual and with worse breathing than usual on the first day of treatment (antibiotics, steroids or both). Four (3.2%) were associated with feeling generally worse than usual alone and 4 (3.2%) with worse breathing than usual alone. Exacerbations in autumn/winter lasted longer than those in spring/summer (median difference of 1.0 day, p = 0.047). Detailed mapping of Bronkotest scores are displayed in Fig 3.

### Urinary biomarkers of AECOPD

In total there were 2052 sets of lateral flow measurements paired with eDiary data for 30 individuals with a median of 71 samples per patient (IQR 40). Reasons for data loss included failure to return the urine cube (n = 10) and insufficient data recorded with the cube (n = 14).

Table 3 presents the concentrations of urinary biomarkers across an exacerbation.

**Table 3:** Urinary concentrations for A1AT, NE, TIMP1, CRP and desmosine over the time course of AECOPD for measured by home lateral flow cytometry.

	-14 to -7 days	-7 to 0 days	0 to 3 days	4 to 7 days	>7 days
n	145	162	110	162	165
A1AT (ng/ml)	50.92 [25.56, 94.73]	55.37 [32.97, 87.08]	67.92 [36.13, 119.30]	59.88 [32.06, 99.65]	58.37 [33.48, 104.97]
NE (ng/ml)	1.00 [0.00, 3.98]	1.74 [0.00, 5.12]	1.59 [0.00, 4.08]	1.24 [0.00, 3.88]	1.30 [0.00, 3.79]
TIMP1 (ng/ml)	6.60 [2.05, 16.09]	6.77 [3.30, 17.40]	5.20 [2.05, 16.12]	5.19 [2.25, 13.55]	8.22 [3.59, 17.48]
CRP (ng/ml)	0.33 [0.22, 2.09]	0.44 [0.21, 3.27]	0.93 [0.26, 4.68]	0.55 [0.19, 3.03]	0.46 [0.20, 2.16]
Desmosine (ng/ml)	100.00 [62.63, 100.00]	100.00 [59.55, 100.00]	100.00 [75.10, 100.00]	100.00 [50.59, 100.00]	100.00 [62.52, 100.00]

Data are expressed as median [1^st^ quartile, 3^rd^ quartile]. A1AT; alpha-1 antitrypsin, NE; neutrophil elastase, TIMP1; tissue inhibitor of metalloproteinases 1, CRP; C-reactive protein. Desmosine assay for all time periods had >50% of values above the threshold limit of 100 ng/ml hence all median values are 100.

For all Anthonisen defined exacerbations urinary CRP was higher during 0 to +3 days (0.93 ng/ml, IQR 4.42) compared to -14 to -7 days (0.33 ng/ml, IQR 1.87, median difference 0.60 ng/ml, p = 0.0004) and subsequently decreased to pre-exacerbation levels after 7 days (0.46 ng/ml, IQR 1.96, p = 0.05). Median urinary CRP was higher in the Anthonisen Type I episodes (1.05 ng/ml, IQR 9.95) compared to Type II/III at 0 to 3 days (0.69ng/ml, IQR 2.32, p = 0.073) and remained higher during 4 to 7 days and >7 days (p = 0.019 and p = 0.001 respectively, S2 Table in S1 File).

There was no detectable exacerbation time course for median concentrations of TIMP1, AAT and NE. Half of all desmosine measurements were at the upper limit of the assay and therefore meaningful analysis could not be performed.

Feasibility of using the urine cube was assessed by thematic analysis of written feedback collected at the end of the study period (S3 Table in S1 File) and visual analogues scores for ease of use. The main themes that emerged included patients forgetting (S2 Fig in S1 File), life events interfering with developing a routine and other health issues/comorbidities. Many noted that performing the test during an acute exacerbation was challenging; “difficult to do when I had a chest infection.” Device led factors mainly related to a poor battery but was considered easy to use as assessed by visual analogue scale (S3 Fig in S1 File).

### AECOPD detection using Bronkotest and urinary biomarkers

To assess predictive ability of the Bronkotest eDiary and urinary biomarkers, ROC analysis was performed for detection of Type I exacerbations against physician review. There were 12,541 days of patient data for either a stable symptom day or the first day of an exacerbation as defined by Anthonisen criteria. First day exacerbations represented 135 entries (1.01%). AUC for change in symptom score (7-day rolling mean) was 0.942 (95% CI 0.900 to 0.984, S4 Fig in S1 File). A threshold symptom score of 6 points gave a sensitivity of 77% and a specificity of 86% for Bronkotest. The predictive performance of the best performing biomarker, CRP, assessed independently and as an addition to Bronkotest symptom scores did not improve the overall predictive ability of eDiary/biomarker combined (AUC 0.818, 95% CI 0.719 to 0.918, S5 Fig in S1 File).

## Discussion

This study is the first to combine daily symptom diary data with regular urine measurement of putative biomarkers of AECOPD in patients with AATD and the largest of treated and untreated exacerbations in AATD.

In general, publications relating to exacerbations in AATD are few compared to those in usual COPD [18]. Data from the current study has shown that symptoms develop rapidly, typically within 48 hours as seen in usual COPD [19]. Treated exacerbations have also been shown to have a higher symptom burden as with usual COPD [19].

Results confirmed Anthonisen Type I exacerbations are longer in duration, are treated more often and have a higher symptom burden, in keeping with a previous study that documented exacerbations in a sub cohort of patients enrolled as part of a randomised controlled trial of inhaled AAT therapy [9]. The burden of untreated exacerbations within frequent exacerbators with AATD related lung disease was also confirmed in a real-world setting where 45% of all Anthonisen Type I exacerbations were untreated. This may be of particular concern in AATD patients who are known to have higher levels of inflammation (and proteinase activity) especially during exacerbations associated with purulent (elastase positive) sputum compared with usual COPD [7]. This could result in short episodes of excessive lung damage [20] hence prompt treatment should be encouraged.

Our results build on findings from Needham et al [21] who identified differences in exacerbation duration and rate occurring in autumn and winter compared with warmer seasons. This could provide evidence for the rationalisation of drugs used to reduce/prevent exacerbations during the summer months in AATD, such as reserving long-term macrolide therapy for winter months only. It also suggests viruses as a factor for exacerbations in AATD related lung disease since these are more prevalent during autumn and winter.

Deriving a Bronkotest score threshold to represent an exacerbation in AATD would enable early recognition of exacerbations in real time. EXACT-PRO [6, 22–24], which uses 2 or 3 days of consecutive increase in symptoms with a difference of 12 or 9 points respectively to define an exacerbation, uses this concept but has only been validated in non-AATD COPD. EXACT-PRO has been approved by regulators as an acceptable outcome suitable for clinical trials in exacerbation studies [25], and having the equivalent simpler but validated tool in AATD would also be useful for future trials of therapies impacting on unreported/untreated episodes [26]. In a real-world setting, meeting a symptom score threshold as well as its characteristics should trigger the patient to seek medical advice and/or take back up therapy [27]. Regardless of the performance of any symptom score, the technology needs to be acceptable to patients in a real-world setting over long periods of time. Our study has provided data supporting feasibility of using an eDiary for up to 18 months although adherence wanes over time.

The use of biomarkers to help strengthen the predictive ability of symptom diaries is an attractive concept. This could define precision medicine [28] in treatment of AECOPD by either alerting patients to a potentially damaging exacerbation or by avoiding oral corticosteroids or antibiotics for episodes that are related to physiological changes alone. Though our results showed some effects on averaged data across the entire cohort during exacerbations, urinary biomarkers were unable to mimic or improve the predictive value of the Bronkotest eDiary. A larger cohort size or better urine cube adherence may have improved the predictability. It was notable that Type I exacerbations were associated with a stronger signal for CRP, supporting the concept that not all exacerbations are associated with increased inflammation [29] and that antibiotics should be aimed at those with purulent sputum. This is especially important in AATD related COPD where increased inflammation from an exacerbation could accelerate emphysema decline [8]. The original Anthonisen randomised control trial found that patients with Type I episodes benefited most from antibiotic treatment compared to placebo [16]. A recent systematic review found moderate evidence that serum CRP was related to bacterial AECOPD [30], though most studies were small with major heterogeneity.

The future of biomarker research currently remains uncertain. The Framington risk score, for example, is successful in predicting future risk of cardiovascular disease at the population level with many biomarkers having strong associations with future cardiovascular events or death [31]. However, the same biomarkers have so far failed to change the AUC to a meaningful extent in prediction models on an individual basis, leading to current pessimism. The same may prove to be the case in AECOPD although more targeted concepts and media remain worthy of further study.

### Strengths and limitations

One of the main strengths of this study is the detail of symptom diary data collected over an extending period of time in a real-world setting. We were able to present average symptom data across the whole course of an exacerbation, as performed previously in studies of COPD but not to a similar extent in AATD [15, 19, 32]. Combining this with urinary biomarkers and a rolling mean to overcome day to day variability was also of merit. Finally, Bronkotest uses a simple scale that changes proportionately to patients symptoms as opposed to the visual analogue score used in EXACT-PRO.

The authors also acknowledge several limitations. This was a single-centre study with a relatively small number of patients which may affect generlisability of results. Some patients failed to engage with the eDiary or urinary monitor leading to missing data and underpowered results, though arguably this reflects real-life. Feasibility data from this study has driven improvements in the urine cube battery life for use in future studies. We were also unable to collect simultaneous sputum samples during exacerbations, since many patients felt too unwell to visit at the time, or were non-productive. This may have helped determine the relevance of bacteria to the episodes as well as the airway inflammation and biomarker profile.

## Conclusion

AECOPD in AATD share some similarities to those observed in usual COPD with a significant burden of unreported exacerbations that may impact health status. Use of an eDiary is feasible to detect and partially characterise such episodes over prolonged periods of time and provide educational patient feedback. This, alongside use of a POC urine device to measure biomarkers of AECOPD, may pave the way for precision medicine. These pioneering results must be validated by more powerful studies.

## Supporting information

S1 Checklist This file contains the STROBE checklist to ensure the manuscript complies with accepted reporting guidelines for observational studies. (DOCX)

S1 File This includes all supplementary methods, figures and tables for this manuscript. (DOCX)
