# Supplementary material for: Characteristics of alpha-1 antitrypsin deficiency related lung disease exacerbations using a daily symptom diary and urinary biomarkers
Source: PLoS One. 2024 Feb 2;19(2):e0297125. doi: 10.1371/journal.pone.0297125 (PMC10836691; doi:10.1371/journal.pone.0297125)
Supplement: S1 Checklist — (DOCX) [file pone.0297125.s001.docx]

STROBE Statement—checklist of items that should be included in reports of observational studies

|  | Item No. | Recommendation | Page  No. | Relevant text from manuscript |
| --- | --- | --- | --- | --- |
| **Title and abstract** | 1 | (*a*) Indicate the study’s design with a commonly used term in the title or the abstract | 1 | were prospectively followed for 18 months |
|  |  | (*b*) Provide in the abstract an informative and balanced summary of what was done and what was found | 1 | See abstract |
| Introduction | | | |  |
| Background/rationale | 2 | Explain the scientific background and rationale for the investigation being reported | 2 | Introduction |
| Objectives | 3 | State specific objectives, including any prespecified hypotheses | 3 | Introduction: final paragraph;” aimed to characterise AECOPD in …AATD related lung disease”, “explore the relationship of exacerbations with changes in urinary biomarkers”, “determine if enhanced monitoring could accurately predict exacerbations.” |
| Methods | | | |  |
| Study design | 4 | Present key elements of study design early in the paper | 3 | See methods – first sentence |
| Setting | 5 | Describe the setting, locations, and relevant dates, including periods of recruitment, exposure, follow-up, and data collection | 3 | Secondary paragraph |
| Participants | 6 | (*a*) *Cohort study*—Give the eligibility criteria, and the sources and methods of selection of participants. Describe methods of follow-up  *Case-control study*—Give the eligibility criteria, and the sources and methods of case ascertainment and control selection. Give the rationale for the choice of cases and controls  *Cross-sectional study*—Give the eligibility criteria, and the sources and methods of selection of participants | 3 | Methods – first paragraph; . “Each patient had a primary diagnosis of COPD confirmed with spirometry (FEV1/FVC ratio <0.7/LLN) and received standard COPD care in line with the GOLD strategy.(1) Patients with a history and diagnosis of asthma were excluded” |
|  |  | (*b*) *Cohort study*—For matched studies, give matching criteria and number of exposed and unexposed  *Case-control study*—For matched studies, give matching criteria and the number of controls per case |  |  |
| Variables | 7 | Clearly define all outcomes, exposures, predictors, potential confounders, and effect modifiers. Give diagnostic criteria, if applicable | 4 | No comparisons made, but criteria for exacerbations given in methods “Anthonisen defined” |
| Data sources/ measurement | 8* | For each variable of interest, give sources of data and details of methods of assessment (measurement). Describe comparability of assessment methods if there is more than one group | *4* | All data was self collected from eDiary or from clinic visits. Urine cube data was collected from POC lateral flow devices issued to the patients. |
| Bias | 9 | Describe any efforts to address potential sources of bias | 4 | No direct comparison used so low risk of bias |
| Study size | 10 | Explain how the study size was arrived at | Supplementary methods page 1 | Sample size calculations were based on unpublished data in usual COPD which required between 88 and 120 Anthonisen defined Type I exacerbations to detect a difference in urinary desmosine. With 80% power and α=0.05, sufficient data collection would be achieved with 55 patients if 60% of patients continued to have 2 to 3 exacerbations per year over 18 months with allowance for 10% drop out |

Continued on next page

| Quantitative variables | 11 | Explain how quantitative variables were handled in the analyses. If applicable, describe which groupings were chosen and why | 4 to 5 | Described in Statistiscal Analysis section in detail |
| --- | --- | --- | --- | --- |
| Statistical methods | 12 | (*a*) Describe all statistical methods, including those used to control for confounding | 4 to 5 | As above |
|  |  | (*b*) Describe any methods used to examine subgroups and interactions | NA | NA |
|  |  | (*c*) Explain how missing data were addressed | 4 | Imputation algorithm included in Data analysis section for eDiary data. |
|  |  | (*d*) *Cohort study*—If applicable, explain how loss to follow-up was addressed  *Case-control study*—If applicable, explain how matching of cases and controls was addressed  *Cross-sectional study*—If applicable, describe analytical methods taking account of sampling strategy |  |  |
|  |  | (*e*) Describe any sensitivity analyses | NA | NA |
| Results | | | | |
| Participants | 13* | (a) Report numbers of individuals at each stage of study—eg numbers potentially eligible, examined for eligibility, confirmed eligible, included in the study, completing follow-up, and analysed | 5 | Consecutive patients enrolled only, no record of those asked who declined so unable to construct flow diagram of eligible participants |
|  |  | (b) Give reasons for non-participation at each stage |  | As above |
|  |  | (c) Consider use of a flow diagram |  | Not able to construct as above |
| Descriptive data | 14* | (a) Give characteristics of study participants (eg demographic, clinical, social) and information on exposures and potential confounders | 5 and Table 1 | Table 1 |
|  |  | (b) Indicate number of participants with missing data for each variable of interest | NA | No missing data at baseline as small group with complete dataset |
|  |  | (c) *Cohort study*—Summarise follow-up time (eg, average and total amount) | 5 | Median follow up of 13.6 months (IQR 7.1) |
| Outcome data | 15* | *Cohort study*—Report numbers of outcome events or summary measures over time | *6* | *271 exacerbations and 13 unclassified exacerbations* |
|  |  | *Case-control study—*Report numbers in each exposure category, or summary measures of exposure |  |  |
|  |  | *Cross-sectional study—*Report numbers of outcome events or summary measures |  |  |
| Main results | 16 | (*a*) Give unadjusted estimates and, if applicable, confounder-adjusted estimates and their precision (eg, 95% confidence interval). Make clear which confounders were adjusted for and why they were included | NA | NA |
|  |  | (*b*) Report category boundaries when continuous variables were categorized | NA | NA |
|  |  | (*c*) If relevant, consider translating estimates of relative risk into absolute risk for a meaningful time period | NA | NA |

Continued on next page

| Other analyses | 17 | Report other analyses done—eg analyses of subgroups and interactions, and sensitivity analyses | 7 | ROC curve analysis and description of biomarkers presented in remaining sections of results |
| --- | --- | --- | --- | --- |
| Discussion | | | | |
| Key results | 18 | Summarise key results with reference to study objectives | 7 to 9 | Discussion |
| Limitations | 19 | Discuss limitations of the study, taking into account sources of potential bias or imprecision. Discuss both direction and magnitude of any potential bias | 9 | Strengths and limitations section included |
| Interpretation | 20 | Give a cautious overall interpretation of results considering objectives, limitations, multiplicity of analyses, results from similar studies, and other relevant evidence | 7 to 9 | Discussed throughout section |
| Generalisability | 21 | Discuss the generalisability (external validity) of the study results | 9 | Needs further studies to validate |
| Other information | |  | | |
| Funding | 22 | Give the source of funding and the role of the funders for the present study and, if applicable, for the original study on which the present article is based | 9 | Alpha1 foundation |

*Give information separately for cases and controls in case-control studies and, if applicable, for exposed and unexposed groups in cohort and cross-sectional studies.

**Note:** An Explanation and Elaboration article discusses each checklist item and gives methodological background and published examples of transparent reporting. The STROBE checklist is best used in conjunction with this article (freely available on the Web sites of PLoS Medicine at http://www.plosmedicine.org/, Annals of Internal Medicine at http://www.annals.org/, and Epidemiology at http://www.epidem.com/). Information on the STROBE Initiative is available at www.strobe-statement.org.
