# Supplementary material for: Characteristics of alpha-1 antitrypsin deficiency related lung disease exacerbations using a daily symptom diary and urinary biomarkers
Source: PLoS One. 2024 Feb 2;19(2):e0297125. doi: 10.1371/journal.pone.0297125 (PMC10836691; doi:10.1371/journal.pone.0297125)
Supplement: S1 File — (DOCX) [file pone.0297125.s002.docx]

Online supplement eDiary paper

# Methods

Sample size calculations were based on unpublished data in usual COPD which required between 88 and 120 Anthonisen defined Type I exacerbations to detect a difference in urinary desmosine. With 80% power and α=0.05, sufficient data collection would be achieved with 55 patients if 60% of patients continued to have 2 to 3 exacerbations per year over 18 months with allowance for 10% drop out.

Table S1: Bronkotest domains and responses with corresponding weighting and symptom score

| Question/statement | Responses | Weighting | Score |
| --- | --- | --- | --- |
| Describe your breathing | Better than usual | 5 | 1 |
|  | Normal/usual | 5 | 2 |
|  | Worse than usual | 5 | 3 |
|  | Much worse than usual | 5 | 4 |
| What colour is your sputum? (colour chart displayed on eDiary) | No sputum | 1 | 0 |
|  | Colour 1 (Clear) | 1 | 1 |
|  | Colour 2 (Mucoid) | 1 | 2 |
|  | Colour 3 (Purulent) | 1 | 3 |
|  | Colour 4 (Purulent) | 1 | 4 |
|  | Colour 5 (Purulent) | 1 | 5 |
| The amount of sputum you produced? | None | 1 | 0 |
|  | A little | 1 | 1 |
|  | Some | 1 | 2 |
|  | Moderate | 1 | 3 |
|  | A lot | 1 | 4 |
| Type of sputum | Watery | 1 | 1 |
|  | Sticky liquid | 1 | 2 |
|  | Semi solid | 1 | 3 |
|  | Solid | 1 | 4 |
| How do you feel? | Better than usual | 5 | 1 |
|  | Normal/usual | 5 | 2 |
|  | Worse than usual | 5 | 3 |
|  | Much worse than usual | 5 | 4 |
| How often do you cough? | Rarely | 1 | 1 |
|  | Occasionally | 1 | 2 |
|  | Frequently | 1 | 3 |
|  | Persistently | 1 | 4 |
| Do you have chest pain? | No | 1 | 0 |
|  | Yes | 1 | 1 |
| Do you have cold or flu symptoms? | No | 1 | 0 |
|  | Yes | 1 | 1 |
| How did you sleep? | Slept well | 1 | 1 |
|  | Woke once | 1 | 2 |
|  | Woke twice | 1 | 3 |
|  | Woke more than twice | 1 | 4 |

Table S2: Urinary biomarkers across the time course of an exacerbation for Anthonisen Type I and Type II/III exacerbations.

| Type 1 | Type I - 0 to 3 days | Type I - 4 to 7 days | Type I - >7 days | *P* |
| --- | --- | --- | --- | --- |
| n | 90 | 96 | 112 |  |
| AAT | 64.05 [36.11, 128.57] | 58.86 [29.57, 88.72] | 63.16 [36.46, 111.50] | 0.434 |
| HNE | 1.12 [0.00, 4.22] | 1.12 [0.00, 3.91] | 2.51 [0.00, 3.81] | 0.912 |
| TIMP1 | 4.35 [1.74, 15.63] | 4.22 [1.50, 10.59] | 8.83 [4.39, 18.51] | <0.001 |
| CRP | 1.05 [0.29, 10.24] | 0.73 [0.24, 4.40] | 0.69 [0.28, 3.64] | 0.472 |
| Desmosine | 100.00 [78.97, 100.00] | 100.00 [44.14, 100.00] | 100.00 [70.81, 100.00] | 0.228 |
| Type II/III |  | | | |
|  | Other - 0 to 3 days | Other - 4 to 7 days | Other - >7 days | *P* |
| n | 58 | 66 | 53 |  |
| AAT | 71.83 [36.95, 115.29] | 61.34 [37.39, 114.26] | 51.96 [29.84, 80.94] | 0.575 |
| HNE | 2.11 [0.00, 3.72] | 1.62 [0.00, 3.68] | 0.00 [0.00, 3.64] | 0.242 |
| TIMP1 | 8.03 [2.27, 16.42] | 7.32 [2.75, 16.67] | 7.58 [3.22, 12.45] | 0.945 |
| CRP | 0.69 [0.23, 2.54] | 0.36 [0.16, 1.82] | 0.27 [0.16, 1.02] | 0.058 |
| Desmosine | 100.00 [74.90, 100.00] | 100.00 [59.20, 100.00] | 100.00 [48.37, 100.00] | 0.461 |

Table S3: Themes identified in patient feedback of urine device use split by visual analogue score (VAS) for frequency of urine device use.

|  | VAS ≥6 (better compliance) | VAS <6 (worse compliance) |
| --- | --- | --- |
| Forgetting | ‘Work’  ‘Routine difficult when working’  ‘Easier to remember when part of a routine’  ‘Stuck to routine’  ‘Did it on a Saturday mostly’  ‘Busy life’  ‘Forgot the rules [of when to test]’ | ‘Fibro [Fibromyalgia] fog’  ‘Busy lifestyle’  ‘Difficult to keep up habit’  ‘Bad memory’  ‘Could use text reminders to use urine test’  ‘Used phone reminder’ |
| Urine cube issues | ‘Battery life very poor’ | ‘Battery an issue [on urine cube]’  ‘Gave up on [the urine cube] as it kept saying ‘error’’  ‘Date kept changing’  ‘The testing kit didn’t work reliably, and I didn’t have time [to repeat the test]’  ‘Sometimes urine did not soak up’,  ‘Sometimes [urine cube] didn’t work properly’.  ‘The odd error was time consuming’.  ‘10 minutes [for it to read]’ |
| Comorbidities/illness | ‘Difficult to do when I had a chest infection’ | ‘Breathlessness; had to use bottle by the side of my bed’ |
| Other | ‘My wife reminds me to do the test’ | ‘Family problems’  ‘Bereavement’  ‘Anxiety’  ‘Tiredness’  ‘Inconvenience’ |


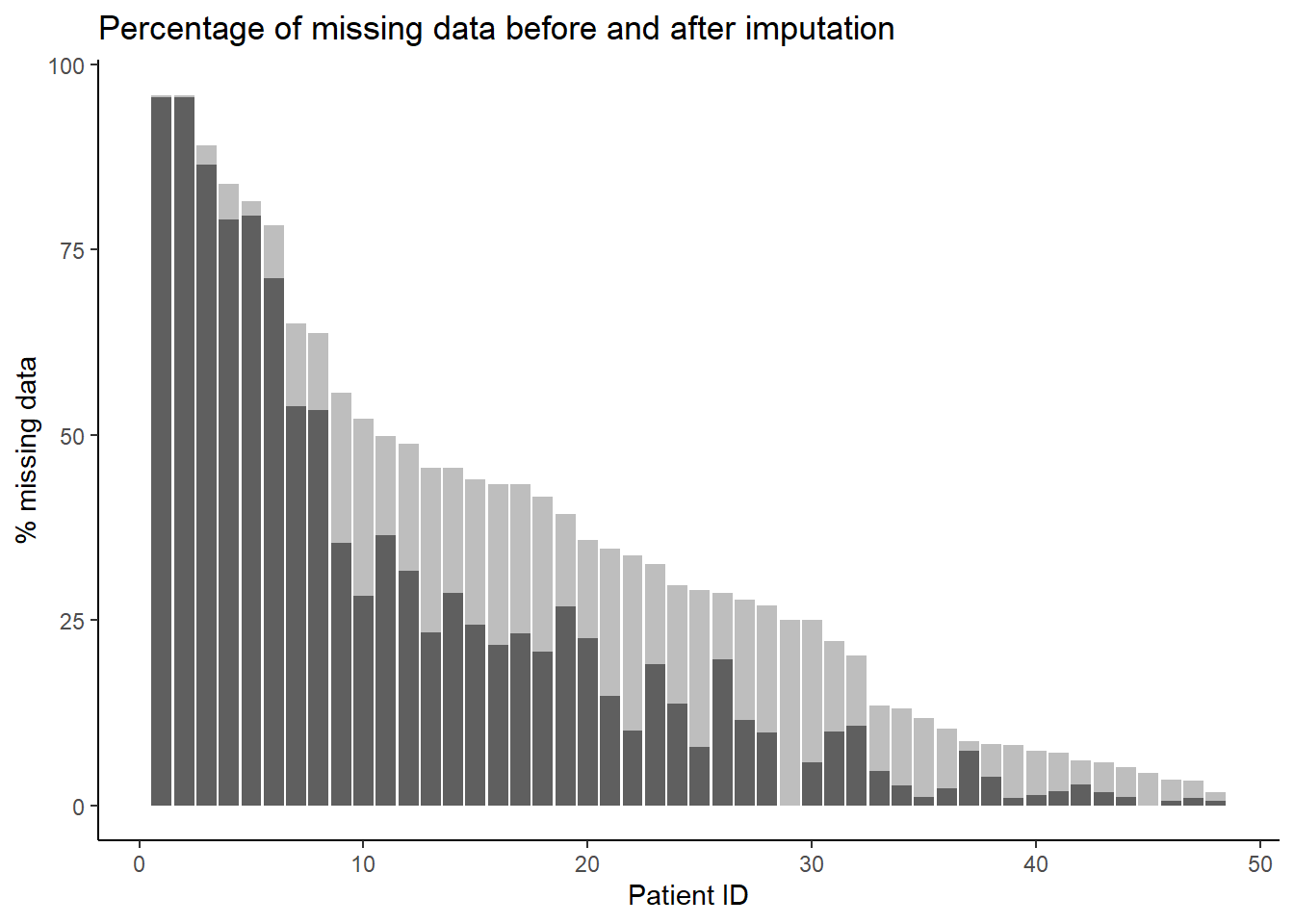


Before Imputation

After Imputation

*Figure S1: percentage of missing data per patient before and after imputation*

*
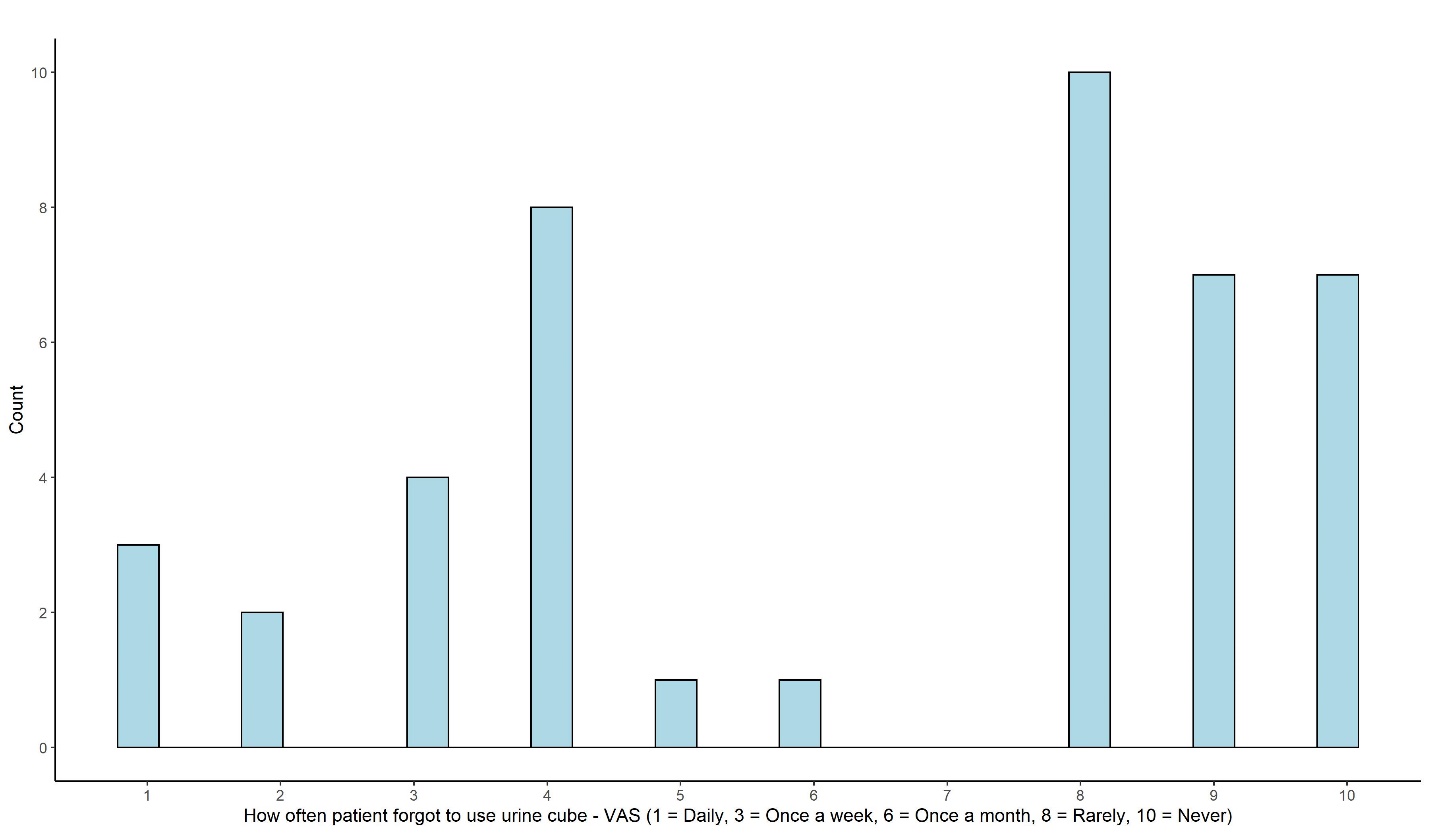
*

Figure S2: Visual analogue score (VAS) of how often patients reported forgetting to use the urine cube.


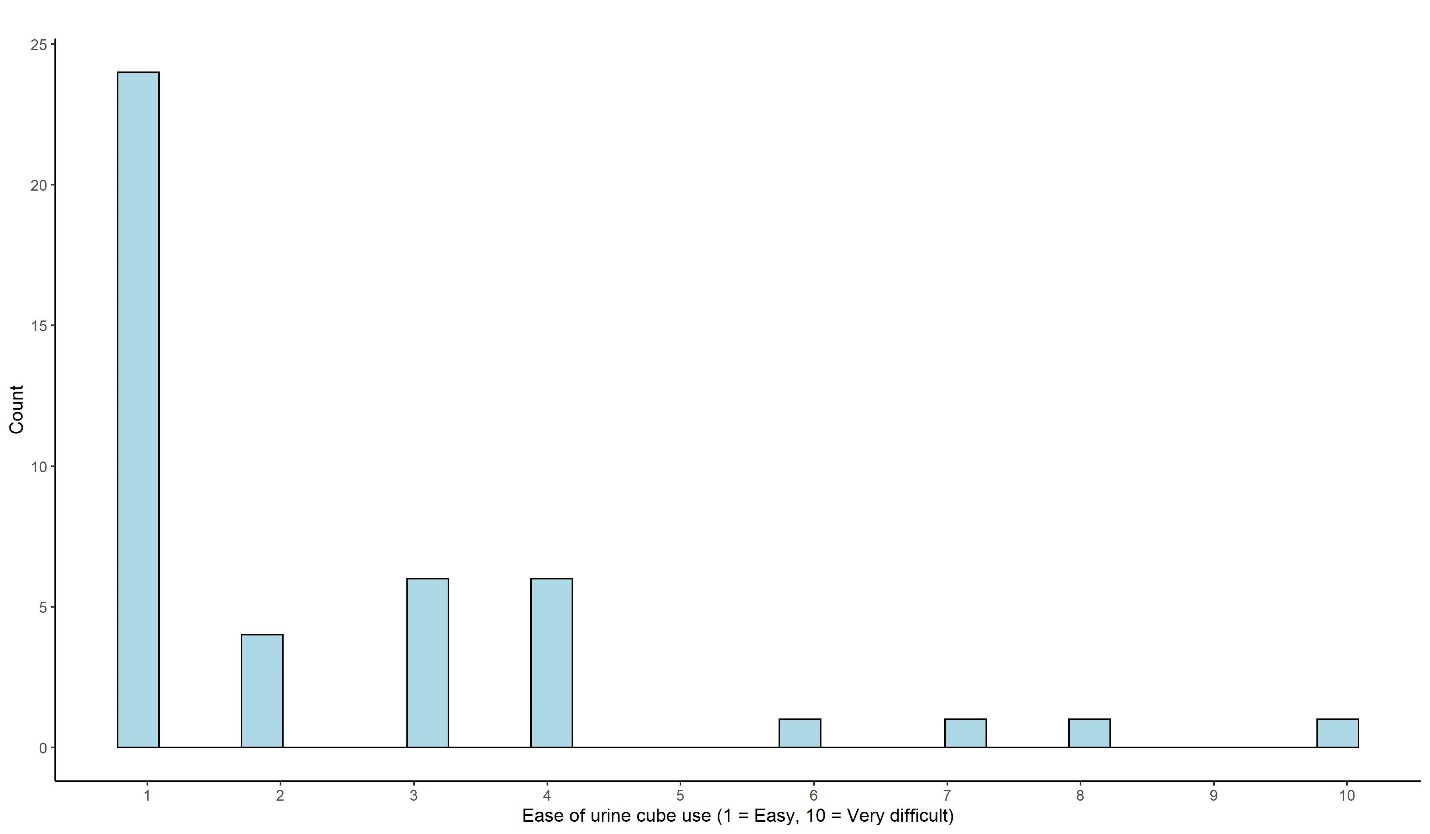


Figure S3: Ease of use of urine cube rated at the end of the study.


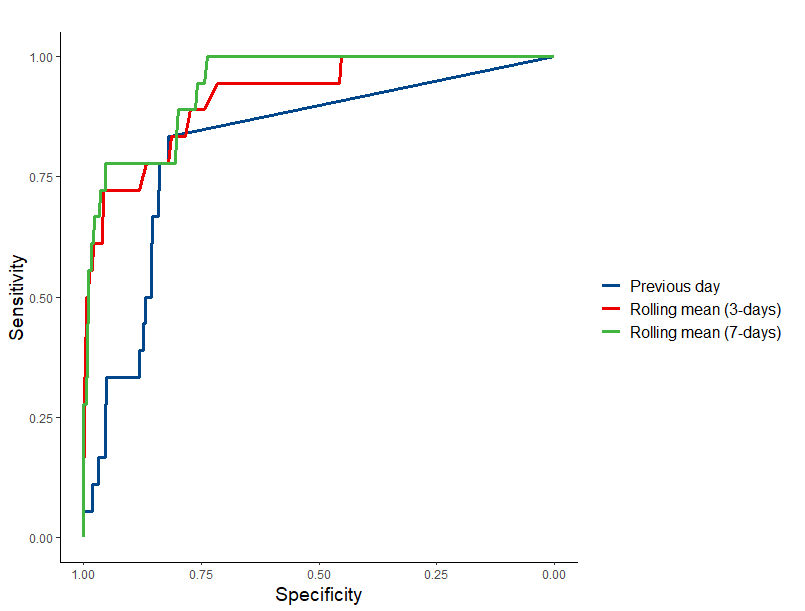


Figure S4: Comparison of different methods of calculating change in symptom score. Previous day AUC 0.818 (95% CI 0.726 to 0.910), Rolling mean (3-days) AUC 0.918 (95% CI 0.851 to 0.985), Rolling mean (7-days) AUC 0.942 (95% CI 0.900 to 0.985).


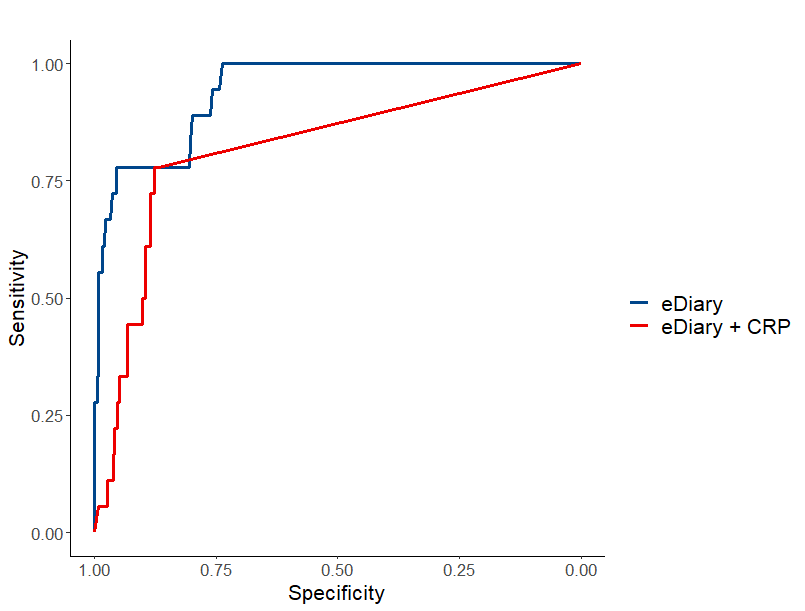


Figure S5: ROC curve comparing predictive ability of the Bronkotest eDiary symptom score alone (AUC 0.942 (95% CI 0.900 to 0.985) and eDiary with urinary CRP (AUC 0.818, 95% CI 0.719 to 0.918).
